# Predictors of mental health problems in formal and informal caregivers of patients with Alzheimer’s disease

**DOI:** 10.1186/s12888-020-02822-7

**Published:** 2020-09-04

**Authors:** Anna Sołtys, Ernest Tyburski

**Affiliations:** 1 Institute of Psychology, University of Szczecin, 69 Krakowska str, 71-017 Szczecin, Poland; 2 Institute of Psychology, SWPS University of Social Sciences and Humanities, 10 Kutrzeby str, 61-719 Poznan, Poland

**Keywords:** Sense of coherence, Generalized self-efficacy, Social support, Mental health, Alzheimer’s disease, Burden of care

## Abstract

**Background:**

Caring for a person with Alzheimer’s disease (AD) is associated with significant mental burden e.g., depression and anxiety, and difficulties with social, familial, and professional functioning. To date, few studies have examined variables which would allow for a comprehensive and detailed study of the relationship between personal resources and caregiver health status, with a majority of studies focusing on factors that contribute to increased caregiver’s burden. Moreover, the available evidence fails to address differences in the functioning of formal and informal carers. Paying proper attention to the problems of nursing home staff can help identify important risk factors. Therefore, this study compared mental health problems in informal and formal caregivers and examined the relationship between mental resources and mental health problems in both groups of caregivers.

**Methods:**

This cross-sectional study examined 100 formal (*n* = 50) and informal (*n* = 50) caregivers of AD patients. Personal resources were measured with the Social Support Questionnaire (SSQ), the Generalized Self-Efficacy Scale (GSES), and the Sense of Coherence Questionnaire (SCQ), while mental health was assessed with the Depression Assessment Questionnaire (DAQ) and the General Health Questionnaire (GHQ). Multivariate stepwise regression was performed separately for both investigated groups.

**Results:**

There were no significant differences between informal and formal caregivers in terms of psychological variables, i.e., sense of coherence, social support, self-efficacy, or mental health problems. In contrast, there were different significant predictors of mental health problems in both groups. Comprehensibility (SCQ) was a significant predictor of mental health problems measured by DAQ and self-efficacy (GSES) was a significant predictor of mental health problems measured by GHQ in informal caregivers. For formal caregivers, emotional support (SSQ) and comprehensibility (SCQ) were significant predictors of mental health problems measured by DAQ, while tangible support (SSQ) and meaningfulness (SCQ) were significant predictors of mental health problems measured by GHQ.

**Conclusions:**

Personal resources are significant predictors of mental health outcomes in caregivers of AD patients. Preventive actions should therefore include assessment of factors affecting caregivers’ mental health in order to provide them with necessary care and create appropriate support groups.

## Background

The progression of Alzheimer’s disease (AD) is associated with significant deficits in patient functioning, necessitating the provision of long-term care. The specific symptoms of Alzheimer’s disease are: increasing cognitive impairment associated with memory deficits, difficulty speaking or recognizing objects, impaired movement control, and behavioral disorders [1]. A recent meta-analysis confirmed that disturbed sleep or unhealthy sleep patterns, including shorter sleep duration and fragmented sleep, altered circadian rest/activity patterns, and insomnia were associated with elevated risk of cognitive impairment, preclinical diagnosis of AD, and AD [2].

Ensuring long-term care for AD patients is largely the responsibility of formal and informal carers. Care is a dynamic process between the caregiver and the patient. Informal care is rooted in the family structure (loved ones, friends, neighbours), while formal care refers to carers who provide it as part of their work or public sector activities [3]. Local associations and centers working for people with Alzheimer’s disease and their families include support groups and educational programs that raise public awareness diseases of old age and organize various forms of assistance for the elderly and their families. The education of carers and the training of medical staff plays an important role. Information exchange and cooperation with domestic and foreign centers also play an important role [4]. Care for patients with Alzheimer’s disease takes place in family homes, nursing homes, day psychogeriatric departments, nursing homes, and private nursing homes.

The concept of caregiver burden, introduced in the 1980s, refers to the emotional, material, social, and physical strain or load borne by the caregiver of a chronically ill person [5]. The intensity of care is related to the magnitude of health effects. Feelings of anxiety and depression have a negative impact on the physical health of the carer. Elevated depression and stress rates as well as reduced subjective well-being in caregivers are caused by the AD patient’s behavioral problems, cognitive impairment, and functional disability, and are related to the duration of care and the caregiver’s age [6].

Behavioral disorders as well as psychotic and affective symptoms in care recipients have been identified as key factors that have an adverse effect on the health of caregivers and their sense of burden [7]. The results of a meta-analysis carried out by [8] indicate a relationship between behavioral and psychological symptoms of dementia and increased burden of care, caregiver depression, the mental health of carers, and elevated patient institutionalization rates. Research by [9] suggests that anxiety is prevalent among carers. In addition to increased caregiver depression and trauma, [10] also reported increased anger, which can significantly reduce the quality and adequacy of provided care.

The physical health of caregivers and their coping strategies indicate increased levels of anxiety [9]. High levels of anger associated with moderate or severe depression lead to a significant increase in potentially harmful behavior in the relationship between caregiver and care recipient [10].

There is a significant increase in levels of stress, depression, and fatigue - manifested through a sense of powerlessness. Greater depressive symptoms and distress from patient problem behaviors predict the onset of cardiovascular disease in caregivers within 18 months of starting care [11]. The length of care influences immune response dysregulation in caregivers, which can persist for up to four years after the death of the care recipient. Caregivers are more likely to develop respiratory infections, suffer from obesity, and have elevated serum lipid levels - contributing to cardiovascular disease. Participation in self-help groups has beneficial effects; it performs an educational and supportive function, which may improve both the psychological and physical conditions of carers and increase their quality of life [12].

One study on a group of 295 participants showed that carers had, on average, 23% higher levels of stress hormones than the control group. These hormones are secreted in large quantities in stressful situations, leading to increased blood glucose [13].

To explain the impact of stressful experiences on caregivers’ physical and mental health, the role of social support should be considered. Support has a direct impact on health. People who experience support are healthier and have a greater sense of belonging, which improves their biopsychosocial functioning. The stress-buffering hypothesis suggests that social support can buffer against stress through a changed perception of competences in an individual struggling with a difficult situation. People who experience support have a greater sense of resourcefulness, which leads to a change in their assessment of their situation and an increase in self-esteem, thus fostering more effective coping with crises [13].

Social support has a significant impact on reducing distress experienced in connection with caring activities. It can be considered an important resource for improving caregiver functioning. Operating as a buffer, social support may reduce the negative effects of stressful situations and severity of fatigue symptoms, thus protecting against the onset of chronic fatigue [12]. Studies indicate that it is a key factor in lowering the level of tension and stress in caregivers [14, 15], contributing to improving physical and mental health and reducing the level of mortality [16].

In health psychology, an important determinant of health behavior is the concept of self-efficacy. It is one of the psychological resources that are responsible for sense of well-being, readiness to make decisions, motivation, and perseverance in achieving goals. Sense of self-efficacy strengthens resilience, increases commitment, and improves coping with difficulties [17].

Optimistic self-beliefs affecting the process of goal setting operate in two phases (pre- and post-intention) and include action self-efficacy (important for action initiation, strategy selection, and outcome expectancies) and coping self-efficacy (associated with optimistic beliefs about one’s own abilities and the ability to foster positive emotions [18];. A higher sense of perceived self-efficacy and better coping skills enhance motivation and performance attainments, which play a significant role in reducing negative health consequences in difficult situations [19]. The role of self-efficacy in shaping mental health should also be mentioned. Caregivers with low self-efficacy show more difficulty in adapting to care requirements. Lack of faith in one’s abilities leads one to focus on the negative aspects of care [20].

Self-efficacy plays a key role in how AD caregivers assess their own health and cope with distress or breakdowns in their normal functioning. It can thus be an important predictor of carers’ mental health.

In the salutogenic model, sense of coherence is a crucial concept that affects an individual’s health and well-being. It refers to an individual’s global orientation concerning their surrounding reality, related to the predictability and rationality of the world [21].

Caring for a chronically ill person is associated with experiencing severe stress, which may lead to the mobilization of numerous resources in an attempt to preserve one’s health [22]. Various studies indicate the existence of an inverse relationship between the sense of coherence and the burden of care [23, 24]. Sense of coherence has also been reported to significantly affect perception of one’s own health [25], the development of depressive symptoms [26], and mortality rates [27]. A strong sense of coherence increases the ability to select coping strategies in a more effective, flexible, and adaptive fashion [21]. Consequently, people with lower sense of coherence are poorer at coping in difficult situations, have unhealthier lifestyles, and report deterioration in their psychosomatic health [28]. Research indicates that a sense of coherence plays a significant role in reducing the sense of burden [19, 29, 30]. A high sense of coherence is associated with reduced risk of various health problems and is considered the main factor associated with the ability to cope with stress [30]. As a crucial resource, sense of coherence can therefore be treated as an important predictor of mental well-being in caregivers of people with Alzheimer’s disease.

To date, few studies have examined variables which would allow for a comprehensive and detailed study of the relationship between personal resources and caregiver health status, with a majority of studies focusing on single factors that contribute to increased caregiver’s burden (e.g., higher sense of coherence is associated with a reduced risk of various health problems and is considered the main factor associated with the ability to cope with stress; caregivers with low self-efficacy have more difficulty adapting to care requirements; social support is a key factor for lowering levels of tension and stress in caregivers, contributing to improving physical and mental health and reducing mortality). Moreover, the available evidence fails to address differences in the functioning of formal and informal carers. Focusing on problems that arise among nursing home staff could help identify important risk factors. Research results remain conflicting. There is a paucity of reports that take into account the impact of various resources on the psychosocial functioning of caregivers. In light of the above, the aim of this study was to compare mental health problems in informal and formal caregivers and examine the relationship between mental resources and mental health problems in both groups of caregivers.

## Methods

### Participants

This study included 100 carers of people with Alzheimer’s disease, divided into two groups based on the nature of their relationship with the patient. The informal carers group consisted of 50 persons recruited at local support centers and institutions. The formal caregivers group consisted of 50 employees of help centers providing care for AD patients. The mean age was *M* = 58.76 for informal caregivers and *M* = 52.92 for formal caregivers. All surveyed caregivers agreed to participate in the study. The study was approved by the Ethics Committee of Institute of Psychology at University of Szczecin (KB 2/2017). Written informed consent was obtained from all participants.

Inclusion criteria were: 2 years minimum duration of care, provision of care for a minimum of 8 h per week, care for patients with Alzheimer’s disease, being aged from 18 to 80 years, and giving informed consent to participate in the study. Exclusion criteria were: death of the care recipient, being aged below 18 years, and caring for patients with other types of dementia.

### Assessment of mental health problems

To measure mental health problems in caregivers, two psychological tools were used: the Depression Assessment Questionnaire (DAQ) - Polish version and the General Health Questionnaire (GHQ) - Polish version. DAQ was used to measure depressive mental health problems. This is a 75-item questionnaire, with each item rated on a 4-point scale. The items reflect basic depressive symptoms, such as: pessimism, guilt, loss of energy, anhedonia, and suicidal ideation. The scores are recorded on five scales: cognitive deficits and energy loss (CDEL); thoughts about death, pessimism, and alienation (TDPA); guilt and anxiety (GA); psychosomatic symptoms and loss of interests (PSLI); and self-regulation (SR). Most of the DAQ scales have high or very high reliability (Cronbach’s alpha ratios from 0.70 to 0.97). The DAQ overall score is characterized by very high reliability (0.95 to 0.97 [31];). Most scales had good reliability in this study (Cronbach’s alpha ratios in entire sample from 0.71 to 0.92) and overall score in entire sample (0.93). GHQ was used to measure general mental health problems. This is a 28-item questionnaire used to assess mental health. It is sensitive to short-term psychiatric disorders, i.e. it identifies people whose mental state has temporarily deteriorated due to experiencing a difficult situation. In addition to the overall score, the questionnaire contains five scales: somatic symptoms, anxiety, insomnia, functional disorders, and depressive symptoms. Based on Cronbach’s alpha index, the internal consistency of the GHQ scale was 0.97 [32]. This scale had good reliability in the present study (Cronbach’s alpha ratio in entire sample 0.92).

### Assessment of psychological resources

To measure psychological resources in caregivers, three psychological tools were used: the Sense of Coherence Questionnaire (SCQ) - Polish version, the Generalized Self-Efficacy Scale (GSES) - Polish version, and the Social Support Questionnaire (SSQ) - Polish version.

SCQ was used to measure quality of life. This is a 29-item questionnaire, with each statement rated on a 7-point scale. The scores are presented on three scales: comprehensibility, manageability, and meaningfulness. The higher the score, the higher the level of sense of coherence. The Polish version of the SCQ has high reliability (Cronbach’s alpha equal to 0.78 [21];). This scale had good reliability in this study (Cronbach’s alpha ratio in entire sample 0.75). GSES was used to measure self-efficacy. The GSES is a 10-item psychometric scale designed to assess optimistic self-beliefs concerning one’s ability to cope with a variety of difficult demands in life. The total score, ranging from 10 to 40 points, is a general indicator of self-efficacy. The higher the score, the greater the sense of self-efficacy. Cronbach’s alpha is 0.85 [33]. This scale had good reliability in the present study (Cronbach’s alpha ratio in entire sample 0.81). SSQ was used to measure social support. The questionnaire is a measure of the central aspects of perceived social support: a subjective belief in the support of others or the availability thereof. The scores are presented on three scales: emotional support (ES), tangible support (TS), and social integration (SI). The Polish version of the questionnaire showed a very high reliability (Cronbach’s alpha of 0.91 [34];). This scale had good reliability in the present study (Cronbach’s alpha ratio in entire sample 0.88).

### Assessment of controlled variables

In this study design, we also considered controlled variables, including: age, sex and education of the caregivers, duration of care, time spent caring per week, support from others, and the stage of Alzheimer’s disease. Data were based on a detailed interview with each participant.

### Statistical analysis

Statistical analysis of the collected data was conducted using the IBM SPSS 25 Statistical package. Sample size was calculated using G-Power software for sample size calculation (two-sample *t*-test and regression analysis). For *t*-tests, we assumed a power of 0.90 and α = 0.05 to detect a medium effect size (0.60) for differences in psychological variables between two groups. According to this calculation, the minimum number of participants was 49 for each of the subgroups, so 98 participants in total. For regression analysis, we assumed a power of 0.90 and α = 0.05 to detect a medium effect size (0.20) for 7 predictors. According to this calculation, the minimum number of participants was 45 in total [35]. Continuous variables are presented in the form of means (*M*) and standard deviations (*SD*). The normality of variable distributions was verified using the Shapiro-Wilk test. To assess the frequency distributions of the categorical variables, we used cross tabulation and the chi-square test. To check for differences between the two groups, we used Student’s *t* test. Cohen’s *d* was used to determine the magnitude of effect size measures. We used Pearson’s *r* to assess the strength of the identified correlations between continuous variables and Cramer’s *V* to calculate correlations between categorical variables and continuous variables. In the case of significant correlations, multivariate stepwise regression was applied separately for each of the two groups.

## Results

### Participant characteristics

Statistical analysis showed that compared to formal caregivers, informal caregivers were older (*p* < 0.05) and had cared for AD patients for longer periods of time since diagnosis (*p* < 0.001), but tended to provide fewer hours of care per week (*p* < 0.01). In the group of informal caregivers, there were more males (*p* < 0.01), more persons with post-graduate education, and fewer persons with only high school education (*p* < 0.01). Moreover, fewer informal caregivers received help from others or got support from various sources (*p* < 0.001), they also provided care to AD patients in the second and third stage of the disease more frequently than patients in the first stage (*p* < 0.05). All demographic and key characteristics are presented in Table 1.

**Table 1:** Demographic characteristics and key variables for informal and formal caregivers

	Informal caregivers (*n* = 50)		Formal caregivers (*n* = 50)			
Variable	*M*	*SD*	*M*	*SD*	*t*	*d*
Age of caregivers	58.76	16.23	52.92	8.95	2.23*	0.45
Duration of care	6.61	4.93	3.66	2.91	3.64***	0.74
Time spent caring per week	26.68	6.39	30.96	6.39	−3.35**	0.68
Proportions					χ^2^	
Caregiver sex						
Female	33^a^		45^b^		8.39**	-
Male	17^a^		5^b^			
Caregiver education						
Primary school graduate	2^a^		1^a^			
Vocational school graduate	2^a^		4^a^		12.97**	-
High school graduate	14^a^		30^b^			
University graduate	32^a^		15^b^			
Help from others						
None	14^a^		2^b^			
From one source	22^a^		15^a^		20.02***	-
From many sources	13^a^		33^b^			
Unknown	1^a^		0^a^			
Stages of Alzheimer’s disease						
First stage	13^a^		24^b^			
Second stage	25^a^		19^b^		5.04*	-
Third stage	12^a^		7^b^			

*Note*. *d* = Cohen’s *d* for effect size measures; *M* = mean; *SD* = standard deviation; *t* = Student’s *t* test; χ^2^ = chi-squared test

* *p* < 0.05. ** *p* < 0.01. *** *p* < 0.001

### Comparison of psychological variables

There were no significant differences between informal caregivers and formal caregivers in terms of psychological variables, i.e. sense of coherence, social support, self-efficacy, depressive symptoms, or mental health problems. Descriptive statistics are presented in Table 2.

**Table 2:** Psychological characteristics of formal and informal caregivers

Variable	Informal caregivers (*n* = 50)		Formal caregivers (*n* = 50)		*t*	*d*
	*M*	*SD*	*M*	*SD*		
SCQ Comprehensibility	48.98	8.23	49.12	6.66	−0.09	–
SCQ Manageability	48.64	7.51	47.26	7.18	0.94	–
SCQ Meaningfulness	39.08	6.24	38.24	6.24	0.67	–
SSQ Emotional support	27.02	4.83	26.98	3.85	0.05	–
SSQ Tangible support	32.50	5.77	33.30	4.82	−0.75	–
SSQ Social integration	27.60	4.92	28.78	4.69	−1.23	–
GSES Self-efficacy	28.92	3.96	28.56	5.76	0.36	–
DAQ Depressive Symptoms	141.46	20.24	138.50	20.51	0.73	–
GHQ Mental Health Problems	29.60	15.01	25.62	15.67	1.30	–

*Note*. *d* = Cohen’s *d* for effect size measures; *M* = mean; *SD* = standard deviation; *t* = Student’s *t* test

*DAQ* Depression Assessment Questionnaire, *GHQ* General Health Questionnaire, *GSES* Generalized Self-Efficacy Scale, *SCQ* Sense of Coherence Questionnaire, *SSQ* Social Support Questionnaire

**Fig. 1:**
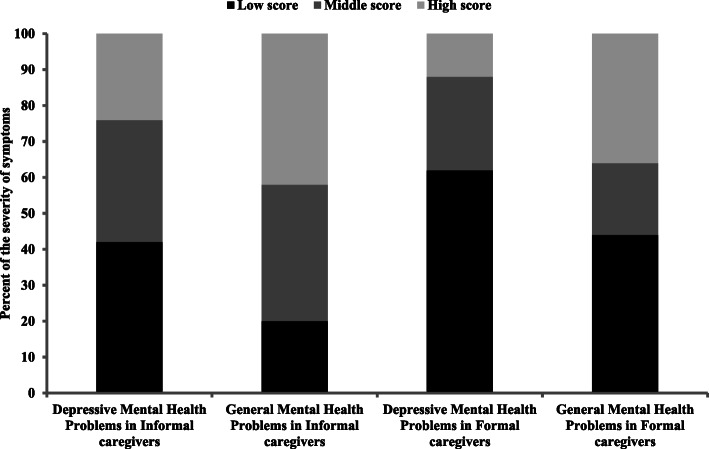
Severity of depressive and general mental health problems in formal and informal caregivers. Low score = score between 1 and 4 sten; middle score = score between 5 and 6 sten; high score = score between 7 and 10 sten

However, as presented in Fig. 1, informal caregivers scored higher (between 7 and 10 sten) on depressive symptoms (24%) and mental health problems (42%) than their formal counterparts (12 and 36%, respectively).

### Predictors of mental health problems

Statistical analyses showed significant negative correlations between comprehensibility (*r* = − 0.56; *p* < 0.001), manageability (*r* = − 0.48; *p* < 0.001), meaningfulness (*r* = − 0.44; *p* = 0.001), self-efficacy (*r* = − 0.39; *p* = 0.005), emotional support (*r* = − 0.38; *p* = 0.006), and depressive mental health problems in informal caregivers.

As presented in Table 3, further multivariate stepwise regression showed that only comprehensibility was a significant predictor of depressive mental health problems in this group, explaining 30% of the variance (the model was well suited to the analysed data, *p* < 0.001). Other independent variables - manageability, meaningfulness, emotional support, and self-efficacy - were excluded from the statistical model.

**Table 3:** Predictors of depressive mental health problems in formal caregivers and informal caregivers

Predictors	β	*t*	*R*^*2*^	*F*
Informal caregivers				
SCQ Comprehensibility	−0.56	−4.68***	0.30	21.90***
SCQ Manageability	0.01	0.06		
SCQ Meaningfulness	−0.01	−0.05		
SSQ Emotional support	−0.21	−1.68		
GSES Self-efficacy	−0.17	−1.27		
Formal caregivers				
SSQ Emotional support	−0.40	−3.13**	0.45	21.02***
SCQ Comprehensibility	−0.38	−3.00**		
SCQ Manageability	0.15	0.62		
SCQ Meaningfulness	0.36	1.39		
SSQ Tangible support	−0.08	−0.42		
SSQ Social integration	−0.17	− 0.86		
GSES Self-efficacy	−0.03	−0.22		

*Note.* For informal caregivers, the following variables were excluded from the statistical model: SCQ Manageability, SCQ Meaningfulness, SSQ Emotional support, and GSES Self-efficacy. For formal caregivers, the following variables were excluded from the statistical model: SCQ Manageability, SCQ Meaningfulness, SSQ Tangible support, SSQ Social integration, and GSES Self-efficacy

*GSES* Generalized Self-Efficacy Scale, *SCQ* Sense of Coherence Questionnaire, *SSQ* Social Support Questionnaire, *β* standardized regression coefficient, *t* Student’s *t* test to determine the significance of a standardized regression coefficient; *R*^*2*^ = size of the explained variance of the dependent (explained) variable including the correction for the number of subjects and the number of predictors introduced into the regression equation; *F* = fit of the regression model

** *p* < 0.01. *** *p* < 0.001

In the group of formal caregivers, the statistical analyses showed significant negative correlations between depressive mental health problems and emotional support (*r* = − 0.61; *p* < 0.001), comprehensibility (*r* = − 0.60; *p* < 0.001), social integration (*r* = − 0.58; *p* < 0.001), manageability (*r* = − 0.55; *p* < 0.001), meaningfulness (*r* = − 0.54; *p* < 0.001), tangible support (*r* = − 0.53; *p* < 0.001), and self-efficacy (*r* = − 0.50; *p* < 0.001).

As presented in Table 3, further multivariate stepwise regression showed that emotional support and comprehensibility were significant predictors of depressive mental health problems in this group, explaining 45% of the variance (the model was well suited to the analysed data, *p* < 0.001). Other independent variables - manageability, meaningfulness, tangible support, social integration, and self-efficacy - were excluded from the statistical model.

Statistical analyses showed significant negative correlations between self-efficacy (*r* = − 0.36; *p* = 0.009), comprehensibility (*r* = − 0.32; *p* = 0.023), meaningfulness (*r* = − 0.31; *p* = 0.028), and general mental health problems in informal caregivers.

As presented in Table 4, further multivariate stepwise regression showed that only self-efficacy was a significant predictor of general mental health problems in this group, explaining 12% of the variance (the model was well suited to the analysed data, *p* < 0.01). Other independent variables - comprehensibility and manageability - were statistically excluded.

**Table 4:** Predictors of general mental health problems in formal and informal caregivers

Predictors	β	*t*	*R*^*2*^	*F*
Informal caregivers				
GSES Self-efficacy	−0.36	−2.71**	0.12	7.35**
SCQ Comprehensibility	−0.20	−1.31		
SCQ Manageability	−0.15	−0.89		
Formal caregivers				
SCQ Meaningfulness	−0.38	−2.76**	0.34	13.70***
SSQ Tangible support	−0.31	−2.27*		
SCQ Comprehensibility	−0.13	−0.48		
SCQ Manageability	−0.02	−0.06		
SSQ Emotional support	0.11	0.51		
SSQ Social integration	0.18	0.65		
GSES Self-efficacy	0.12	0.73		

*Note.* For informal caregivers, the following variables were excluded from the statistical model: SCQ Comprehensibility and SCQ Manageability. For formal caregivers, the following variables were excluded from the statistical model: SCQ Comprehensibility, SCQ Manageability, SSQ Emotional support, SSQ Social integration, and GSES Self-efficacy

*GSES* Generalized Self-Efficacy Scale, *SCQ* Sense of Coherence Questionnaire, *SSQ* Social Support Questionnaire, *β* standardized regression coefficient, *t* Student’s *t* test to determine the significance of a standardized regression coefficient; *R*^*2*^ = size of the explained variance of the dependent variable (explained) including the correction for the number of subjects and the number of predictors introduced into the regression equation; *F* = fit of the regression model

* *p* < 0.05. ** *p* < 0.01. *** *p* < 0.001

In the group of formal caregivers, there were significant negative correlations between general mental health problems and comprehensibility (*r* = − 0.49; *p* < 0.001), manageability (*r* = − 0.47; *p* = 0.001), meaningfulness (*r* = − 0.52; *p* < 0.001), self-efficacy (*r* = − 0.36; *p* = 0.009), emotional support (*r* = − 0.47; *p* = 0.001), tangible support (*r* = − 0.55; *p* < 0.001), and social integration (*r* = − 0.48; *p* < 0.001).

As presented in Table 4, further multivariate stepwise regression showed that tangible support and meaningfulness were significant predictors of general mental health problems, explaining 34% of the variance (the model was well suited to the analysed data, *p* < 0.001). Other independent variables - comprehensibility, manageability, emotional support, social integration, and self-efficacy - were statistically excluded.

## Discussion

To the best of our knowledge, this is the first study to systematically examine the association between mental resources (self-efficacy, sense of coherence, and social support), and mental health problems (understood as two psychological dimensions: depressive mental health problems and general mental health problems) in formal and informal caregivers. We found that personal resources are significant predictors of both dimensions of mental health outcomes in both groups of carers.

The purpose of this research was to understand how psychosocial factors modify the mental health of carers of people with Alzheimer’s disease. This paper is based on theoretical background and empirical research on the psychosocial functioning of caregivers. We provide a description of caregivers’ mental health, expressed by the level of depression and assessment of their general health. Our results present certain predictors of mental health in carers of AD patients.

As stated in the introduction, the role of a carer of an AD patient is a difficult task, often extending beyond one’s adaptive capabilities, and is frequently associated with experiencing chronic stress [36]. Many studies suggest the existence of links between the severity of the disease and negative changes in the psychosomatic health of the carers, as well as increased morbidity and mortality [6, 37].

Our results indicate that a poor sense of comprehensibility is an important predictor of depressive mental health problems in formal and informal carers. As a component of the sense of coherence, this variable is associated with the perception of both external and internal stimuli as coherent and understandable [21]. A low sense of comprehensibility may result in a belief that the caregiver’s role is complicated, chaotic, inexplicable, or difficult to sort out. Hence, they may find it difficult to organize, explain, and find meaning in their situation [38].

A strong sense of comprehensibility, on the other hand, correlates with absence of depressive symptoms. It enhances the adaptation of personal resources to external contextual requirements, promoting a proactive attitude towards encountered difficulties. Such findings are consistent with the available research, which shows that the important predictors of depression in carers of people with Alzheimer’s disease are factors related to caregiver characteristics and psychological situation, including their ability to cope with stress, sense of coherence, social support, personality, and the ages and financial status of the caregiver and care-recipient [39]. Studies also suggest that caregivers with a lower sense of coherence are at a higher risk of experiencing an increased burden of care as well as developing both physical and mental health problems [40].

Our research also indicates the importance of emotional support in the group of formal carers. Availability of supportive emotional behaviors - associated with a sense of acceptance from others and the ability to share feelings, care, and compassion - can reduce depressive symptoms. Such results are in line with research indicating the key role of social, family, and institutional support in reducing perceived burden of care [41]. However, in this study, the relationship between emotional support and depression was only significant in the group of formal caregivers. This suggests that emotional support may be a stronger predictor of depressive symptoms among those who perform the caregiver role as part of their professional work. In part, this may result from a greater need for acceptance, understanding, and compassion among formal carers who do not receive compensation for the burden of care they bear as part of their relationship with the care recipient. Nevertheless, the correlation observed in this study requires further investigation.

Our research indicates that the most significant predictor of general mental health problems in the group of informal carers is self-efficacy. Stronger self-efficacy favors greater motivation and commitment to attaining goals, even in situations that pose a major challenge to the individual. It is also conducive to involvement in health-related activities, active coping with stress, overcoming obstacles, and focusing on opportunities [42]. Low self-efficacy increases the risk of anxiety, depression, or feelings of helplessness, which can lead to a deterioration in psychosomatic health [43]. Our findings suggest that the belief that one’s own actions are ineffective may be associated with a stronger subjective experience of stress and lower tolerance of stress. Care for close relatives involves emotional relations, which may increase the sense of control over external events and the sense of responsibility. A perceived inability to cope with difficulties results in a surge in stress levels, which affects activation of the autoimmune system, leading to immune deficiency. Development of a strong sense of self-efficacy may have an immunoenhancing effect [44]. Therefore, it seems important not only to have specific psychological resources, but also to perceive one’s own capabilities as sufficient to perform the caregiver role.

In the group of formal carers, the sense of meaningfulness was the strongest predictor of mental health. The higher its level, the better the carers’ perception of their mental health. Sense of meaningfulness is the most important emotional-motivational component of sense of coherence [45]. A strong sense of meaningfulness may be associated with the belief that fulfilling the role of carer is really worthwhile and that there is good reason to commit to it. Formal carers can be protected from burnout syndrome by encouraging them to consider their work a challenge, building greater involvement, and finding meaning in their actions. In the face of difficult events, people with a strong sense of meaningfulness can better understand the situation they are in, are more aware of available coping strategies, and are more motivated to overcome difficulties [38]. Studies on persons with somatic illness indicate that sense of coherence is a strong determinant of positive health outcomes and successful coping [28]. The sense of meaning plays a major role here, reinforcing the other two elements [21], and constituting an important resource for maintaining proper health levels [45].

The results of our analyses also indicate the importance of tangible support in the group of formal carers. A perceived availability of help in dealing with problems at work and relief from excessive duties can lead to a better assessment of health. Various studies suggest that support can reduce negative health outcomes resulting from performing a caregiver role [46, 47]. Working with the elderly can be a great burden, which can lead to burnout. The burden is related to, inter alia, the patient-caregiver relationship that is created during the provision of care. Caregivers who maintain better relationships with patients are more effective in their work [48]. In turn, caring for a patient with no emotional attachment can lead to excessive criticism, anger, and hostility. Absence of sufficient support, social isolation, and stigmatization tend to result in an elevated sense of burden and greater feelings of loneliness. All this may lead to a decreased quality of life [49].

Comprehensive research into the relationship between personal resources and caregivers’ health, with a particular focus on the differences between formal and informal care, can help assess relevant risk factors. The statistical analyses performed showed no significant differences in the levels of personal resources, depressive mental health problems, or general mental health problems in carers from the two groups examined, which may be an important point for further analysis of problems in psychosocial functioning, especially in the population of formal carers. It seems particularly important to provide emotional and psychological support to caregivers to prevent professional burnout as well as to include caregivers who are at the very start of their careers. There are several limitations when interpreting the results of this study. Cross-sectional analyses preclude the determination of causal associations. Increasing the sample size would allow for more advanced analyses that could demonstrate other types of dependencies. It seems important to focus on the needs of caregivers and the possibility of introducing preventive measures aimed at building personal resources, which could foster better coping with the burden of care. We were also limited by the base study measures. The GHQ is a screening tool that does not allow clinical assessment of mental health. Another important limitation was the lack of a stress assessment tool. Nevertheless, this study provides the foundation for subsequent work to elucidate knowledge of the proliferation of caregiving stress among aging adults caring for relations with functional disability. The last limitation is the lack of data regarding the validity of the psychological tools calculated in this study. However, there is much data about the validity of these tools from other studies and we can assume that they have good psychometric value.

The study showed intergroup differences in gender, education level, sources of support, duration of care, and stage of the disease. As other studies indicate, factors such as gender, education level, number of hours devoted to caring for patients, duration of care, and stage of the disease play an important role in shaping the level of care burden. Studies indicate that women experience a greater sense of care burden [50–52] and are more vulnerable than men to stress and the development of depressive disorders [53–55]. Low levels of education are related to an increase in the occurrence of depression and burden symptoms [56, 57]. Caring for patients at later stages of the disease and longer duration of care are associated with greater burden [58–60], the severity of anxiety and depression [61], and the deterioration of the somatic state of caregivers [62]. Therefore, the obtained test results should be interpreted with great caution due to the differences shown.

Formal carers are a group whose role is to support family carers, supplement care services, and sometimes take over care functions. The daily care of a patient is a source of chronic stress, which can lead to numerous negative outcomes, including negative assessment of one’s own life, emotional distance from the care recipient, neglect, hostility, and indifference to their needs. Fatigue, resulting from too many duties, significantly contributes to the deterioration of psychosomatic health [63]. In this study, there were no statistically significant differences between formal and informal carers in the assessment of mental health, which suggests that significant problems are not limited to family carers only. However, effective action can only be initiated once the problem has been examined in greater detail. This study may thus become the foundation for introducing systematic research, allowing the assessment of factors affecting the mental health of formal and informal carers and providing such carers with adequate support. Organization of caregiver support groups seems to be of particular importance. The use of appropriate psychotherapeutic interventions can improve the psychosomatic health of caregivers.

An increase in self-efficacy can affect an individual’s physiology, contributing to improved health outcomes and sense of well-being. The inclusion of caregivers in a social support network helps them increase their self-esteem, gives a sense of greater opportunities, and fosters positive feelings, thus constituting a protective factor against the development of somatic diseases [64]. Analysis of empirical research by [65] indicates the mediation of the sense of coherence in explaining health. Researchers suggest a positive relationship between the sense of coherence and subjective health assessment [66], and better mental and physical health [67]. Predictors of mental health presented in this paper contribute to the current picture of the problem. Strengthening caregivers’ personal resources can reduce the likelihood of their manifesting non-adaptive behaviors. The proposed model of mental health promotion in caregivers of AD patients is the starting point for more systematic research, the results of which could help refine the model. It is also worth considering the role of other factors that are important for the mental health of carers, such as personality, stress levels, lifestyle, and cognitive functioning.

## Conclusions

In conclusion, this study determines predictors of mental health in formal and informal carers of people with Alzheimer’s disease. Sense of comprehensibility is a key element in the development of depressive mental health problems, alongside emotional support, which is a predictor of depressive mental health problems in the group of formal caregivers. Sense of self-efficacy is important for the assessment of general mental health in the group of informal carers, while in the group of formal carers it is the sense of meaningfulness and tangible support that affect general mental health outcomes. Future research should involve a comprehensive approach to intervention aimed at strengthening caregivers’ personal resources in order to improve their mental health.

## Data Availability

Supporting data are available from the corresponding author.

## Abbreviations

AD: Alzheimer’s disease
CDEL: Cognitive deficits and energy loss
*d*: Cohen’s *d* for effect size measures
DAQ: Depression Assessment Questionnaire
ES: Emotional support
*F*: Fit of the regression model
SSQ: Social Support Questionnaire
GA: Guilt and anxiety
GHQ: General Health Questionnaire
GSES: Generalized Self-Efficacy Scale
*M*: Mean
PSLI: Psychosomatic symptoms and loss of interests
*r*: Pearson’s *r* coefficient correlations
*R*^*2*^: Size of the explained variance of the dependent (explained) variable including the correction for the number of subjects and the number of predictors introduced into the regression equation
SCQ: Sense of Coherence Questionnaire
*SD*: Standard deviations
SI: Social integration
SR: Self-regulation
*t*: Student’s *t* test or Student’s *t* test to determine the significance of a standardized regression coefficient
TDPA: Thoughts about death, pessimism, and alienation
TS: Tangible support
β: Standardized regression coefficient
χ^2^: Chi-squared test
